# Impact of DICER1 and DROSHA on the Angiogenic Capacity of Human Endothelial Cells

**DOI:** 10.3390/ijms22189855

**Published:** 2021-09-12

**Authors:** Heike Braun, Michael Hauke, Anne Ripperger, Christian Ihling, Matthew Fuszard, Robert Eckenstaler, Ralf A. Benndorf

**Affiliations:** 1 Department of Clinical Pharmacy and Pharmacotherapy, Institute of Pharmacy, Martin-Luther-University Halle-Wittenberg, 06120 Halle (Saale), Germany; heike.braun@pharmazie.uni-halle.de (H.B.); michael.hauke@pharmazie.uni-halle.de (M.H.); anne.ripperger@pharmazie.uni-halle.de (A.R.); robert.eckenstaler@pharmazie.uni-halle.de (R.E.); 2 Department of Pharmaceutical Chemistry and Bioanalytics, Institute of Pharmacy, Charles Tanford Center, Martin Luther University Halle-Wittenberg, 06120 Halle (Saale), Germany; christian.ihling@pharmazie.uni-halle.de; 3 Core Facility—Proteomics Mass Spectrometry, Charles Tanford Centre, Martin-Luther-University Halle-Wittenberg, 06120 Halle (Saale), Germany; matthew.fuszard@medizin.uni-halle.de

**Keywords:** endothelial cells, angiogenesis, microRNA biogenesis, DICER1, DROSHA, RNA interference

## Abstract

RNAi-mediated knockdown of DICER1 and DROSHA, enzymes critically involved in miRNA biogenesis, has been postulated to affect the homeostasis and the angiogenic capacity of human endothelial cells. To re-evaluate this issue, we reduced the expression of DICER1 or DROSHA by RNAi-mediated knockdown and subsequently investigated the effect of these interventions on the angiogenic capacity of human umbilical vein endothelial cells (HUVEC) *in vitro* (proliferation, migration, tube formation, endothelial cell spheroid sprouting) and in a HUVEC xenograft assay in immune incompetent NSG^TM^ mice *in vivo*. In contrast to previous reports, neither knockdown of DICER1 nor knockdown of DROSHA profoundly affected migration or tube formation of HUVEC or the angiogenic capacity of HUVEC *in vivo*. Furthermore, knockdown of DICER1 and the combined knockdown of DICER1 and DROSHA tended to increase VEGF-induced BrdU incorporation and induced angiogenic sprouting from HUVEC spheroids. Consistent with these observations, global proteomic analyses showed that knockdown of DICER1 or DROSHA only moderately altered HUVEC protein expression profiles but additively reduced, for example, expression of the angiogenesis inhibitor thrombospondin-1. In conclusion, global reduction of miRNA biogenesis by knockdown of DICER1 or DROSHA does not inhibit the angiogenic capacity of HUVEC. Further studies are therefore needed to elucidate the influence of these enzymes in the context of human endothelial cell-related angiogenesis.

## 1. Introduction

Angiogenesis is defined as a process that leads to the formation of new blood vessels from pre-existing ones. It is tightly regulated and plays an important role not only in the physiological adaptation of the vascular bed to the metabolic demands of the tissue, but also in tissue regeneration after ischemia and in wound healing [1,2]. Furthermore, angiogenesis plays an essential role in the expansion of embryonic and fetal blood vessel networks and contributes significantly to the growth of malignant tumors [1,2]. At the beginning of the angiogenic process, activation of the normally quiescent vascular endothelium mediated by pro-angiogenic signals occurs, which then initiates and facilitates angiogenic sprouting, proliferation, migration, and tube formation of endothelial cells in the surrounding tissue. This ultimately leads to the formation of new vascular structures that are connected to the blood circulation and mature under the mechanical influences of blood flow and through recruitment of mural cells to gain full functionality [1,2]. The process of angiogenesis is governed by an interplay of stimulatory and inhibitory factors, such as vascular endothelial growth factor-A (VEGF-A/VEGF) as one of the central regulators of endothelial differentiation, vascular morphogenesis, as well as physiological and pathophysiological angiogenesis or thrombospondin-1, an endogenous angiogenesis inhibitor, whose expression in endothelial cells may be regulated by specific microRNAs (miRNAs) [3,4,5]. Interestingly, the VEGF-A-VEGFR-2 axis is thought to play an important role not only in new blood vessel formation, but also in the homeostasis of vascular endothelial cells [6]. In addition, it plays a role in the regulation of vascular tone and systemic blood pressure, which are thought to be influenced by VEGF-A-VEGFR-2-mediated activation of Akt-eNOS and thus activation of endothelial survival signaling and nitric oxide release [7].

In addition to the aforementioned factors, the process of angiogenesis is also influenced by certain miRNAs, so-called angiogenic miRNAs (AngiomirRs) [8]. Mature miRNAs are short, highly conserved, non-coding RNAs of approximately 22 nucleotides that play an important role in the complex network of gene regulation, particularly in gene silencing [9]. The genes for miRNAs are encoded in the genome and are transcribed mainly by RNA polymerase II to form so-called primary transcripts (pri-miRNAs). Pri-miRNAs are then processed by DROSHA and the dsRNA binding protein DGCR8 in a nuclear microprocessor complex to yield the approximately 65 nucleotide pre-miRNA, which forms a characteristic hairpin structure [9]. The pre-miRNA is actively exported by exportin-5 in the presence of Ran-GTP as a cofactor across the nuclear pores of the nuclear membrane into the cytoplasm, where it is cut into ds-miRNAs by the enzyme DICER1 (Dicer) [9]. One of the miRNA strands (the guide strand) is usually much more abundant and biologically active, as it is retained in the RNA-induced silencing complex (RISC), which contains an AGO protein that is capable of binding and cleaving mRNAs. In this context, AGO protein guidance to target mRNA sequences via the guide strand contributes to post-transcriptional gene regulation by binding of miRNAs to the 3’ untranslated region (3’-UTR) of the mRNAs, which then are either inhibited from translation, or degraded, depending on the complementarity of the binding sequence and the proteins involved [9].

In the process of angiogenesis, miRNAs seem to be profoundly involved in the regulation of expression of angiogenesis-related factors and, via these, affect important sub steps of angiogenesis, such as angiogenic sprouting, migration, proliferation, and tube formation of endothelial cells [10]. Thus, miRNA expressed in endothelial cells may regulate cell-autonomous behaviour and cell–cell interactions and thus influence blood vessel formation. In addition, miRNAs expressed and secreted by other somatic or malignant tumor cells may act on endothelial cells via microvesicles or by regulating the expression and paracrine action of pro- or anti-angiogenic factors and thus, may affect angiogenesis via these routes [10,11]. Besides altered expression levels of certain miRNAs, it has also been shown that the process of new blood vessel formation can be affected by dysregulation of the miRNA biogenesis enzymes DICER1 and DROSHA, enzymes that also appear to be involved in endothelial cell differentiation and morphogenesis [12,13,14]. In this context, a role of DICER1 in postnatal angiogenesis in the murine organism has also been described [15]. Nonetheless, other groups have postulated that DICER1 may rather act as an anti-angiogenic factor in postnatal angiogenesis through suppression of VEGF-A-related actions or generation of otherwise anti-angiogenic miRNAs [16,17,18]. We therefore re-evaluated the impact of a DICER1 and DROSHA knockdown on the angiogenic capacity of human endothelial cells *in vitro* and *in vivo* and surprisingly found that knockdown of DICER1 and DROSHA, in contrast to previous reports [12,13], does not impair but rather stimulates the angiogenic capacity of human umbilical vein endothelial cells. In line with these findings, DICER1 or DROSHA knockdown only modestly affected the global proteome of human endothelial cells and here, for instance, reduced the expression of anti-angiogenic thrombospondin-1. We therefore conclude that further experimental evidence is needed to elucidate the impact of DICER1 and DROSHA on the angiogenic capacity of vascular endothelial cells, at least in humans.

## 2. Results

### 2.1. Expression of DICER1 and DROSHA in HUVEC and Evaluation of RNAi-Related DICER1 and DROSHA Knockdown Efficiencies

**Figure 1:**
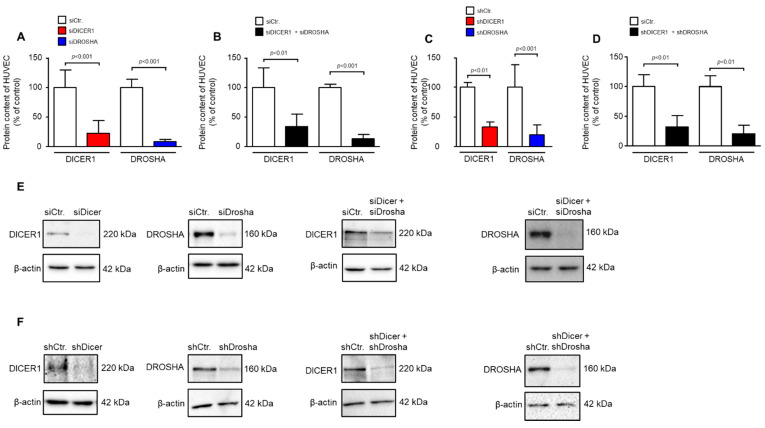
RNAi-mediated knockdown of DICER1 and/or DROSHA in HUVEC significantly reduces DICER1 and/or DROSHA protein content in these cells. (**A**) siRNA-mediated knockdown of either DICER1 or DROSHA or both (**B**) significantly reduces the respective protein content in HUVEC as evidenced by Western Blot detection (*n* = 5–6 biological replicates from independent transfections). (**C**) shRNA-mediated knockdown of either DICER1 or DROSHA (**C**) or both (**D**) significantly reduces the respective protein content in HUVEC as evidenced by Western Blot detection (*n* = 7–9 biological replicates from independent lentiviral transductions). Data are shown as mean ± SD. (**E**,**F**) For each analysis a representative Western Blot is shown for siRNA-mediated (**E**) and shRNA-mediated (**F**) knockdown.

We used transient siRNA or lentiviral stable shRNA delivery to HUVEC to achieve knockdown of DICER1, DROSHA or DICER1 and DROSHA. As determined by real-time PCR analyses, shRNA directed against DICER1 led to a significant reduction in HUVEC DICER1 mRNA content (27.9 ± 12.9%) vs. non-targeting shRNA control (100 ± 35.5%, *p* < 0.05, *n* = 3 biological replicates from independent transductions, Supplemental Figure S1). This was also the case, but only as a non-significant trend, with shRNA directed against DROSHA (45.1 ± 8.9%) as compared to non-targeting shRNA control (100 ± 52.7%, *p* = 0.15, *n* = 3 biological replicates from independent transductions, Supplemental Figure S1). We therefore subsequently investigated the more important effects of RNAi treatment on protein levels of DICER1 and DROSHA in HUVEC. As determined by Western Blot analyses, siRNA directed against DICER1 led to a significant reduction in HUVEC DICER1 protein content (22.4 ± 21.4%) vs. non-targeting siRNA control (100 ± 29.3%, *p* < 0.001, *n* = 5–6 biological replicates from independent transfections, Figure 1A,E). In addition, siRNA-mediated knockdown of DROSHA significantly reduced the content of this protein in HUVEC (8.4 ± 4.0%) as compared with a non-targeting control (100 ± 13.5%, *p* < 0.001, *n* = 6 biological replicates from independent transfections, Figure 1A,E). Moreover, simultaneous knockdown of both DICER1 and DROSHA was achieved with a similar efficacy when both siRNA mixtures were used (Figure 1B,E). In a second approach, stable knockdown of DICER1 and/or DROSHA was induced by lentiviral delivery of specific shRNAs or non-targeting shRNA control. In this approach, DICER1 knockdown led to a significant reduction in HUVEC DICER1 protein content (33.0 ± 19.3%) as compared with non-targeting shRNA control (100 ± 20.2%, *p* < 0.01, *n* = 5–6 biological replicates from independent transductions, Figure 1C,F). In addition, shRNA-mediated knockdown of DROSHA significantly reduced the content of this protein in HUVEC (19.5 ± 16.4%) as compared with non-targeting control (100 ± 38.4%, *p* < 0.001, *n* = 9 biological replicates from independent transductions, Figure 1C,F). Again, simultaneous knockdown of both DICER1 and DROSHA was achieved with a similar efficacy when both shRNAs were used (Figure 1D,F). In each experiment, a small fraction of cells was taken for analyses to verify knockdown at the protein level. Only those experiments were used for analysis in which significant knockdown of DICER1 and DROSHA was detected.

### 2.2. Effect of DICER1 and/or DROSHA Knockdown on the Angiogenic Capacity of HUVEC

#### 2.2.1. Effect of DICER1 and/or DROSHA Knockdown on Angiogenic Sprouting of HUVEC Spheroids

**Figure 2:**
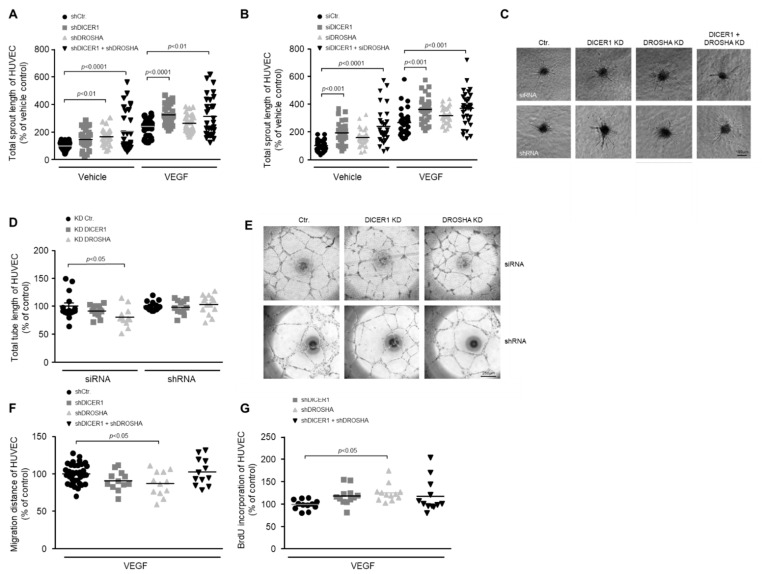
Impact of RNAi-mediated knockdown of DICER1 and/or DROSHA on the angiogenic capacity of HUVEC *in vitro*. RNAi-mediated knockdown of DICER1 as well as DICER1 and DROSHA using either specific shRNA (**A**,**C**); *n* = 30–52) or siRNA (**B**,**C**); *n* = 29–30 biological replicates derived from at least three independent sets of experiments) increases basal and VEGF-induced sprouting from HUVEC spheroids *in vitro*. Data are depicted as scatter plots and mean. (**C**) Representative microscopic pictures of VEGF-induced (20 ng/mL) HUVEC spheroid sprouts. RNAi-mediated knockdown of DROSHA but not DICER1 using either specific shRNA or siRNA may slightly decrease HUVEC migration (**F**); *n* = 12–36 biological replicates derived from at least three independent sets of experiments) and tube formation (**D**,**E**); *n* = 12–15 biological replicates derived from at least three independent sets of experiments), although the siRNA-related knockdown effect on the tube formation capacity of HUVEC could not be observed in HUVEC, in which DROSHA knockdown was performed using shRNA (**D**). (**E**) Representative microscopic pictures of HUVEC tubes. (**G**) shRNA-mediated knockdown of DROSHA but not DICER1 moderately increases BrdU incorporation of HUVEC (*n* = 12 biological replicates derived from at least three independent sets of experiments).

To clarify the role of DICER1 and DROSHA in the process of angiogenic sprouting, we performed single or combined siRNA- or shRNA-mediated knockdown of these genes 24 h prior to generation of HUVEC spheroids and then analysed basal or VEGF-induced angiogenic sprouting of spheroids derived from treated or appropriate control HUVEC. In these experiments we were able to show that knockdown of DICER1 (both siRNA- and shRNA-mediated) enhanced both basal and VEGF-induced angiogenic sprouting, whereas the knockdown of DROSHA did not significantly affect angiogenic sprouting (Figure 2A–C). Moreover, simultaneous knockdown of DICER1 and DROSHA using either specific siRNAs or shRNAs additively and considerably enhanced both basal and VEGF-related angiogenic sprouting from HUVEC spheroids (Figure 2A–C).

#### 2.2.2. Impact of DICER1 and/or DROSHA Knockdown on Tube Formation of HUVEC

To clarify the role of DICER1 and DROSHA in the formation of endothelial tubes, we performed siRNA- and shRNA-mediated knockdown of these genes prior to the *in vitro* analyses. In contrast to angiogenic sprouting, tube formation of HUVEC was not significantly affected by DICER1 knockdown (Figure 2D,E). In addition, it tended to be lower in HUVEC, in which DROSHA knockdown was induced by siRNA but not in HUVEC in which DROSHA knockdown was induced by shRNA (Figure 2D,E). Due to the lack of a clear effect in these experiments, we refrained from a combined knockdown of DICER1 and DROSHA.

#### 2.2.3. Impact of DICER1 and/or DROSHA Knockdown on the Migration of HUVEC

To analyse the influence of DICER1 and DROSHA on the motility of endothelial cells, we performed single or combined shRNA-mediated knockdown of these genes 48 h prior to the *in vitro* analyses in the wound healing assay. In these experiments, migration of HUVEC was not significantly affected by DICER1 or by a combined DICER1 and DROSHA knockdown. However, DROSHA knockdown slightly reduced the motility of HUVEC in this experimental set up (Figure 2F).

#### 2.2.4. Impact of DICER1 and/or DROSHA Knockdown on BrdU Incorporation of HUVEC

To clarify the role of DICER1 and DROSHA in the proliferation of endothelial cells, we performed shRNA-mediated knockdown of these genes prior to the *in vitro* analyses. In these experiments, BrdU incorporation of HUVEC tended to be higher both in DICER1 and in DICER1 and DROSHA knockdown HUVEC, effects that, however, did not reach statistical significance (Figure 2G). Nonetheless, DROSHA knockdown moderately but significantly increased BrdU incorporation of HUVEC in our experimental set up (Figure 2G).

### 2.3. Effect of DICER1 or DROSHA Knockdown on the Angiogenic Capacity of HUVEC in the Xenograft Assay in NSG^TM^ Mice In Vivo

**Figure 3:**
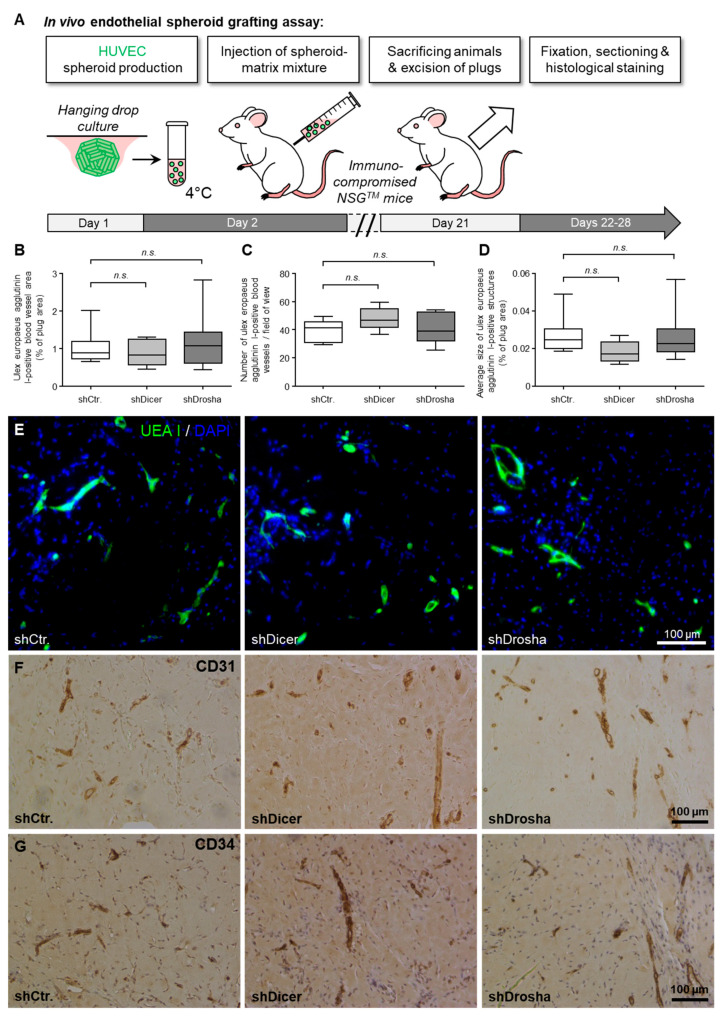
Impact of RNAi-mediated knockdown of DICER1 or DROSHA on the angiogenic capacity of HUVEC *in vivo*. (**A**) Schematic illustration of the experimental setup of the HUVEC spheroid-based grafting assay in immunodeficient NSG^TM^ mice. (**B**–**D**) shRNA-mediated knockdown of DICER1 or DROSHA in HUVEC does not affect VEGF- and bFGF-induced blood vessel growth of these cells *in vivo* as compared to non-targeting shRNA-expressing control HUVEC (*n* = 7–9 xenograft plugs). *n.s.* denotes non-significant. Blood vessel growth was analysed by quantification of the Ulex europaeus agglutinin I-positive blood vessel area (**B**). Moreover, the number of Ulex europaeus agglutinin I-positive blood vessels per high-power field (**C**) as well as the average size of these vessels (**D**) was determined. (**E**) Representative microscopic pictures of xenograft plug sections stained with Ulex europaeus agglutinin I (UEA I) and DAPI (4’,6-diamidino-2-phenylindole) for selective visualisation of the vascular network derived from implanted HUVEC and nuclei, respectively. Representative microscopic pictures of Xenograft plug sections stained with antibodies directed against human endothelial cell markers CD31 (PECAM1; (**F**)) or CD34 (**G**) for further phenotypic characterisation of the vascular network derived from implanted HUVEC.

To clarify whether DICER1 and DROSHA contribute to the angiogenic capacity of human endothelial cells *in vivo*, we used the well-characterised HUVEC xenograft implantation assay [19] in immune-compromised NSG^TM^ mice (Figure 3A). For this assay, we used lentiviral stable shRNA delivery to HUVEC to achieve permanent knockdown of DICER1 or DROSHA or permanent expression of non-targeting shRNA control, respectively. In this experimental set up, neither knockdown of DICER1 nor knockdown of DROSHA significantly affected humanoid vessel formation (mean blood vessel area, mean blood vessel density, mean size of blood vessels; Figure 3B–G) as evidenced by histomorphometric computer-aided analysis of Ulex europaeus agglutinin I-positive blood vessels (Figure 3E). These humanoid Ulex europaeus agglutinin I-positive blood vessels also expressed typical human endothelial cell markers, such as CD31 (PECAM1) and CD34 (Figure 3F,G), indicating that the detected blood vessels were formed from implanted HUVEC.

### 2.4. Effect of DICER1 and/or DROSHA Knockdown on the Proteome of HUVEC

**Figure 4:**
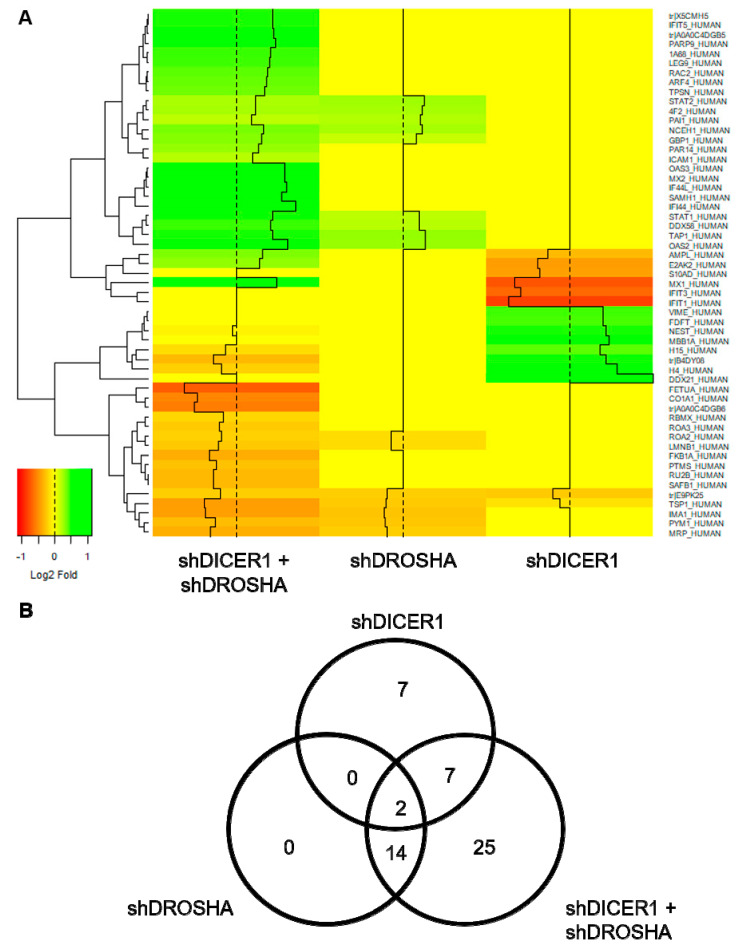
Global proteome analyses of HUVEC engineered to express specific shRNA directed against DICER1, DROSHA or DICER1 and DROSHA or non-targeting shRNA control. (**A**) Heat map visualisation of differentially expressed genes (DEGs) identified in global proteome analyses of HUVEC, in which either DICER1, DROSHA or DICER1 and DROSHA knockdown was performed by stable expression of specific shRNA and compared to HUVEC stably expressing non-targeting shRNA control (*n* = 3 biological replicates derived from independent lentiviral transductions for each experimental condition). The heatmap color range from green to orange represents the log2 fold expression value of control HUVEC in which green denotes genes with high expression levels and orange denotes genes with low expression levels. In these unbiased analyses, for instance, a reduced expression of anti-angiogenic thrombospondin-1 (TSP-1) was observed in DICER1, DROSHA and in particular in DICER1 and DROSHA knockdown HUVEC. (**B**) The number and overlap of DEGs in DICER1, DROSHA and DICER1 and DROSHA knockdown HUVEC is depicted by Venn diagrams.

To assess the effect of DICER1 and DROSHA on HUVEC protein expression, we performed unbiased global proteome analyses 72 h after stable lentiviral shRNA delivery to HUVEC to achieve permanent knockdown of DICER1, DROSHA or both DICER1 and DROSHA. In these analyses, only a low number of differentially expressed genes were identified (Figure 4). Compared with non-targeting shRNA control, both knockdown of DICER1 and knockdown of DROSHA induced deregulation of 16 proteins (Figure 4B), respectively. Moreover, simultaneous knockdown of DICER1 and DROSHA induced deregulation of 48 proteins, although knockdown-associated changes in protein content were consistently moderate to negligible in nature. However, interestingly and in contrast to a previous publication [11], we observed a reduction in the expression level of the angiogenesis inhibitor thrombospondin-1 (TSP-1) in DICER1, DROSHA and even more pronounced in DICER1 and DROSHA knockdown HUVEC, suggesting that both DICER1 and DROSHA regulate TSP-1 expression in HUVEC. In addition, we observed decreased expression of a non-muscle isoform of cofilin-1 (E9PK25) after knockdown of DICER1, DROSHA, and DICER1 + DROSHA. Cofilin-1 regulates actin polymerisation and appears to play an important role in cell motility, cell morphology, and cell division, which may contribute to the observed endothelial phenotype of knockdown of DICER1 and/or DROSHA on the angiogenic capacity of HUVEC. In contrast to the downregulated proteins mentioned above, STAT1 (signal transducer and activator of transcription 1), a known negative regulator of angiogenesis, which functions as a signaling protein and transcription factor, was upregulated by DROSHA as well as by DICER1 + DROSHA knockdown, whereas, in contrast, singular knockdown of DICER1 had no effect on STAT1 expression.

## 3. Discussion

Many experimental studies have shown that the process of angiogenesis can be profoundly affected by epigenetic aspects of gene regulation as well as by regulating the expression of miRNAs, so-called angiogenic miRNAs (AngiomirRs) [8]. For example, miRNAs appear to be critically involved in regulating the expression of angiogenesis-related factors and, through them, influence important sub steps of angiogenesis, such as angiogenic sprouting, migration, proliferation, and tube formation of endothelial cells [10]. In addition, it is conceivable that miRNA expressed in endothelial cells regulate cell-autonomous behaviour and cell–cell interactions and also influence blood vessel formation via these mechanisms. In addition to altered expression levels of certain miRNAs, it has also been demonstrated that the process of new blood vessel formation and the function and angiogenic capacity of human endothelial cells can be modified by RNAi-mediated downregulation of the miRNAs biogenesis enzymes DICER1 and DROSHA, enzymes that may also be involved in endothelial cell differentiation and morphogenesis in the embryonic or fetal organism [12,13,14]. Interestingly, in this context, it has been shown that the enzyme DICER1 also has a role in postnatal angiogenesis in the murine organism [15]. However, the experimental findings of other groups rather suggested that DICER1 might exert anti-angiogenic effects in the process of postnatal angiogenesis by suppressing VEGF-A expression and VEGF-A-induced blood vessel growth [16,18]. Suppression of DICER1 may, for instance, represent a general mechanism to enhance the response of the vascular endothelium to hypoxia. In this context, one publication suggested that chronic hypoxia reduces DICER1 expression and activity both *in vitro* and *in vivo*, with global implications for miRNA biogenesis [17]. In this experimental work, downregulation of DICER1 was induced by von Hippel-Lindau-dependent signaling pathways and was essential for the expression and function of hypoxia-inducible factor (HIF) subunits. Hence, these data indicate that downregulation of DICER1 and suppression of miRNA biogenesis may represent a mechanism that could also serve to induce the angiogenic response of human endothelial cells.

The aim of this work was therefore to clarify the impact of the enzymes DICER1 and DROSHA on the angiogenic capacity of human endothelial cells *in vitro* and *in vivo.* To analyse this matter, we used the same endothelial cell type (HUVEC) and a similar methodology (RNAi-mediated knockdown, *in vitro* and *in vivo* angiogenesis assays) as described in previous studies that have investigated this issue [12,13]. In contrast to these previous publications, however, we surprisingly found that knockdown of DICER1 and DROSHA does not negatively affect the angiogenic capacity of HUVEC. For instance, we were able to show that both DICER1, as well as DICER1 and DROSHA knockdown, tended to induce the BrdU incorporation as evidence for an increased proliferative capacity of HUVEC and significantly enhanced the angiogenic sprouting from HUVEC spheroids *in vitro*. The latter effect was induced similarly by siRNA- and shRNA-mediated knockdown, thereby indicating that specific rather than non-specific “off-target” RNAi-related effects were responsible for the observed changes in the angiogenic phenotype of human endothelial cells. The reasons for these opposing results are unclear. Nevertheless, one can speculate that the ratio of endothelial expression levels of pro-angiogenic versus anti-angiogenic miRNAs must be relevant to predict the effect that disruption of miRNA biogenesis by knockdown of the DICER1 or DROSHA enzymes will have in the endothelial cells studied. In principle, the miRNA expression profile could be influenced by the culture conditions of the cells *in vitro*, which, however, in general is highly standardised across laboratories when using commercially available culture media and additives. On the other hand, it has been shown that (patho)physiological situations that favour angiogenesis, such as low oxygen partial pressures in tissues *in vivo* or hypoxia during tissue ischemia *in vivo*, may induce a profound deregulation of the miRNA expression profiles in vascular endothelial cells [20]. Thus, the *in vivo* environment or pathological situations of tissue ischemia may change the impact that a disruption of miRNA biogenesis could have on the angiogenic capacity of vascular endothelial cells. To exclude potential *in vitro* bias and to study the impact of DICER1 and DROSHA *in vivo*, we therefore also implanted DICER1 or DROSHA knockdown or appropriate control HUVEC into immune-incompetent NSG^TM^ mice and investigated their potential to form humanoid blood vessels in the murine organism. Interestingly, again, there was no detectable inhibitory effect of the knockdown of DICER1 or DROSHA on the angiogenic capacity of implanted human endothelial cells. The reasons for the apparent inconsistencies with respect to data from other published work on this topic [12] are unclear. However, with regard to the *in vivo* data, we would like to point out that, in contrast to Kuehbacher and colleagues [12], we used stable lentiviral gene transfer to express specific shRNAs or appropriate non-targeting shRNA control, a technique that enables long-term knockdown of DICER1 and DROSHA during humanoid blood vessel formation in mice. In our view, this may therefore represent a more appropriate method to analyse the functional significance of the two enzymes *in vivo* than transient transfection of siRNA, which was chosen in the mentioned study [12]. Nevertheless, additional studies are needed to further characterise and elucidate the influence of these enzymes on human endothelial cell-associated angiogenesis.

Besides the functional characterization of HUVEC conducted in this study, we also performed unbiased global proteome analyses, in which we studied the effect of DICER1 and/or DROSHA knockdown on the protein expression profiles of HUVEC. In line with our findings derived from the functional analyses, DICER1 or DROSHA knockdown only modestly affected the protein expression profiles of human endothelial cells. For instance, in contrast to previously published data [12], DICER1 and DROSHA additively reduced (not increased) the expression of anti-angiogenic thrombospondin-1 (TSP-1). Indeed, this effect may explain, at least in part, the rather pro-angiogenic effect of DICER1 and DROSHA downregulation in our experiments, which we observed *in vitro* and which involved an increase in the BrdU incorporation of HUVEC and enhanced angiogenic sprouting from HUVEC spheroids, respectively. In addition to thrombospondin-1, another protein, a non-muscular isoform of cofilin-1 was additively negatively regulated by double knockdown of DICER1 and DROSHA in HUVEC. The protein controls actin filament dynamics [21] and previous reports have suggested that its knockdown modulates endothelial cell proliferation, tip cell formation, and neovascularization processes [22] and thus may be mechanistically related to DICER1- and DROSHA-induced endothelial cell phenotypes. Nevertheless, we have been able to show that pharmacological inhibition of the Rho kinase isoforms ROCK1 and ROCK2, an intervention that also decreases cofilin-1 activity in endothelial cells, can reduce anti-angiogenic stimuli and thus increase endothelial cell motility and tube formation [22]. In this context, it must be mentioned that by disrupting microRNA biogenesis through knockdown of DICER1 and/or DROSHA, suppression of gene expression is not necessarily to be expected, since the repressive effect of various microRNAs on translation or mRNA stability is lost. Nevertheless, knockdown of DICER1 and/or DROSHA in HUVEC in our hands led to a reduction in the expression of some target proteins (e.g., thrombospondin-1 and cofilin-1). On a speculative level, this effect could be mediated by the loss of certain microRNAs, which may lead to increased expression and transrepression activity of inhibitory transcription factors, which in turn could lead to a reduction in the expression of factors such as thrombospondin-1 or cofilin-1 in HUVEC via complex mechanistic interconnections. On the other hand, it is also conceivable that the deletion of DICER1 and DROSHA leads to a reduction in the expression of genes in a microRNA-independent direct or compensatory manner (e.g., signaling molecules, transcription factors), and thus the mechanism is not exclusive to the disruption of microRNA biogenesis. In addition, the signaling molecule STAT1 [23] was upregulated in DROSHA and DROSHA + DICER1 knockdown HUVEC. Activated STAT1 signal transduction in HUVECs has been suggested to suppress genes involved in the response of pro-angiogenic factors like VEGF and bFGF [24], thereby inhibiting their angiogenic response. However, it is not clear weather sole upregulation of STAT1 in HUVEC without its activation induces a similar phenotype. Nevertheless, further analyses in human endothelial cells need to clarify whether the additional silencing of STAT1 has an effect on the DICER1 and DROSHA-mediated phenotype of endothelial cells.

In conclusion, in the present work we observed that neither knockdown of DICER1 nor knockdown of DROSHA profoundly affected proliferation, migration, tube formation or angiogenic sprouting of HUVEC *in vitro*, although previous reports had suggested an essential role of these enzymes for the angiogenic capacity of this cell type. Moreover, we obtained very similar results in a HUVEC xenograft assay in immune incompetent NSG^TM^ mice *in vivo*, in which again neither stable DICER1 nor stable DROSHA knockdown significantly affected humanoid blood vessel formation. In line with our *in vitro* and *in vivo* results, global proteome analyses in HUVEC revealed that knockdown of DICER1 or DROSHA only moderately affected HUVEC protein profiles, but again in contrast to previous publications, additively reduced the expression of the angiogenesis inhibitor thrombospondin-1. We therefore believe that further research is needed to clarify the role of DICER1 and DROSHA in human endothelial cell-related angiogenesis.

## 4. Materials and Methods

All chemicals and reagents were purchased from Sigma-Aldrich (St. Louis, MO, USA), unless stated otherwise. Recombinant human VEGF-A and bFGF were obtained from PeproTech Inc. (Rocky Hill, CT, USA). ON-TARGET PLUS siRNAs directed against DICER1 (L-003483-00-0005) and DROSHA (L-016996-00-0005) were purchased from GE Healthcare Dharmacon (Lafayette, CO, USA). A non-targeting siRNA negative control (SIC001) was obtained from Sigma-Aldrich.

### 4.1. Cell Culture, Transient Transfection and Lentiviral Transduction of Human Endothelial Cells

Human umbilical vein endothelial cells (HUVEC) were purchased from PromoCell and passaged in endothelial cell growth medium (PromoCell, Heidelberg, Germany) on gelatine-coated multi-well plates or cell culture flasks according to the manufacturer’s specifications at 37 °C in humidified air with 5% CO_2_. HUVECs in passages 2–5 were used for the experiments. For transient transfection, HUVEC were detached and transiently transfected with non-targeting siRNA negative control or specific siRNAs (all at 500 nmol/L) against DICER1 or DROSHA, respectively, using HUVEC-specific transfection kits and the nucleofector™ 2b device (all from Lonza, Basel, Switzerland) according to the instructions of the manufacturer. Afterwards, transfected cells were seeded in gelatin-coated 6-well plates at a density of 500,000 cells per well prior to functional- and/or gene expression analyses. Transduction of HUVEC was performed with VSV-G-pseudotyped lentiviral particles derived from the GIPZ™ backbone (Horizon Discovery; Waterbeach, UK). For shRNA-mediated knockdown of DICER1 (V2LMM_30829) and DROSHA (V2LMM_10811), commercially available shRNA sequences, as well as a non-silencing shRNA control (RHS4346) pre-cloned into the GIPZ™ backbone were used (Horizon Discovery, Waterbeach, UK). psPAX2 (#12260) and pMD2.G (#12259) plasmids were obtained from addgene (Cambridge, MA, USA). For stable lentiviral delivery of specific shRNAs and non-targeting shRNA control, HUVECs were infected with a multiplicity of infection (MOI) of 100. Infected cells were cultured for 48 to 72 h prior to functional or gene expression analyses depending on the experimental set up. In each experiment, a small fraction of cells was taken for analyses to verify knockdown at the protein level. Only those experiments were used for analysis in which significant knockdown of DICER1 and DROSHA was detected. Lentiviral transduction efficiencies were additionally determined by detecting GFP as a co-expressed reporter protein in endothelial cells. Efficiencies usually exceeded 90%.

### 4.2. Production, Purification and Titer Determination of Lentiviral Vectors

Production, purification and titration of lentiviral vectors was performed as described by Kutner et al. [25] In short, HEK293T cells were seeded in 150 cm^2^ dishes at a density of 8 × 10^6^ cells/dish in DMEM high-glucose medium supplemented with 10% FCS, 1% penicillin-streptomycin mix and 1% GlutaMAX (all from Life Technologies, Carlsbad, CA, USA). After 24 h, cells were used for transfection at a density of approximately 40%. 6 mL transfection mix per dish contains the transfer vector (60 µg), the VSV-G envelope-expressing plasmid pMD2.G (21 µg), the second-generation lentiviral packaging plasmid psPAX2 (39 µg), 2 M CaCl_2_, water and 2 × HEPES-buffered saline (Life Technologies) at pH 7.07. Chloroquine at a final concentration of 25 µmol/mL was added to the medium right before transfection. Medium was changed once 20 h post-transfection. Two days after transfection, the cell supernatant was collected, centrifuged (500× *g*, 10 min) and filtered (pore size 0.45 µm). The filtered supernatant was mixed in centrifuge beakers with 50% polyethylene glycol 6000, 4 M NaCl and 1 × phosphate-buffered saline. The mixture was stored at 4 °C for 90 min and the beakers were shaken every 30 min. To concentrate the lentiviral particles, suspension was centrifuged (7000× *g*, 10 min, 4 °C) and the pellet was resuspended in 50 mM Tris-HCl, pH 7.4. Lentiviral preparations were stored at −80 °C.

Lentiviral vector titers were determined by flow cytometry (Attune^®^ Acoustic Focusing flow cytometer, Thermo Fisher Scientific, Waltham, MA, USA). For this, 3 × 10^4^ HUVECs per well were plated in gelatine-coated 12-well plates (Greiner, Tokyo, Japan) and transduced with certain amounts of virus suspension. 48 h post-transduction with known amount of virus suspension, cells were harvested and washed with 1 × PBS/10% FCS. The fraction of eGFP-positive cells was measured by flow cytometry [26]. The virus titer was calculated according to the equation: Transducing Units (TU) mL^−1^ = (F × D × N)/V, where F is the percentage of GFP-positive cells, D is the fold dilution of virus used for transduction, N is the number of cells at the time of transduction and V is the volume of diluted virus added per well at transduction.

### 4.3. Angiogenic Sprouting (Endothelial Cell Spheroid) Assay

The potential of angiogenic sprouting from endothelial spheroids was analysed as described by Korff and Augustin [27]. In brief, HUVEC were harvested and suspended in endothelial growth medium with 20% methocel (methyl cellulose in endothelial growth medium). Endothelial aggregates (spheroids) containing 400 cells each, were formed in ’hanging drops’ overnight in non-adherent culture-dishes at 37 °C in humidified air with 5% CO_2_. Afterwards, 50 spheroids were embedded in 0.5 mL of embedding matrix consisting of equal amounts of rat collagen and methocel with 20% FCS in non-adhesive 48-well plates (Greiner). Spheroids were kept in basal endothelial medium (PromoCell, Beijing, China) with VEGF (20 ng/mL) for 24 h at 37 °C in humidified air with 5% CO_2_. Spheroids and their sprouts were visualised using an epifluorescence microscope (Nikon, Minato City, Tokyo). Angiogenic sprouting was quantified by measuring the cumulative sprout length of each spheroid using the NIS elements digital software (Nikon). At least three independent sets of experiments were performed. The biological replicates of each set were then normalised to control to allow for combined statistical analysis.

### 4.4. Endothelial Tube Formation Assay

The organisation of HUVEC into capillary-like networks (tube-formation) was assessed as described before [28] by using growth factor-reduced matrigel (Corning Life Science, Tewksbury, MA, USA). In brief, matrigel was polymerised at 37 °C for 30 min in 96-well angiogenesis µ-plates (10 µL/well, Ibidi). Then 1.5 × 10^4^ HUVEC were seeded per well and incubated in basal endothelial medium (PromoCell) supplemented with 2% FCS at 37 °C, humidified air and 5% CO_2_ for 24 h. Tube length and morphological changes were visualised using an epifluorescence microscope (Nikon). Total tube length was measured with 10× magnification and NIS elements software (Nikon). At least three independent sets of experiments were performed. The biological replicates of each set were then normalised to control to allow for combined statistical analysis.

### 4.5. Migration Assay

Directional migration of endothelial cells was analysed via the scratch wound assay [29]. Briefly, 35,000 cells/well were seeded in a gelatine-coated 48-well plate, transduced with lentiviral vectors and grown to confluence for 72 h. Then, a wound was set creating a gap in which cells could migrate. During the course of the experiment, cells were incubated in basal endothelial growth medium supplemented with 2.5% FCS. Cell movement was imaged at 20 min intervals for up to 24 h using the CFI Plan Apochromat 10× objective. All analyses of time-lapse image series, including migration distance and speed, were performed using the NIS elements software package (Nikon). At least three independent sets of experiments were performed. The biological replicates of each set were then normalised to control to allow for combined statistical analysis.

### 4.6. Endothelial Cell Proliferation Assay

The proliferation rate of endothelial cells was analysed by the BrdU (5-bromo-2′-deoxyuridine) incorporation assay. Incorporation of BrdU in de-novo-synthesised DNA was investigated using a commercially available cell proliferation ELISA (Roche, Basel, Switzerland) according to the manufacturer’s instructions. In brief, 2500 endothelial cells per well were plated in a gelatine-coated 96-well plate (Greiner) and transduced with the appropriate lentiviral vector. 72 h post-infection, cell growth medium was supplemented with BrdU (final concentration 100 µM) for 3 h at 37 °C, humidified air and 5% CO_2_. Afterwards, cells were fixed, denatured and incubated for 90 min with BrdU antibody, following the manufacturer’s instructions. Finally, stained cells were washed once with 1× PBS and peroxidase substrate solution was added. Read-out at 450 nm was done after stopping the peroxidase reaction with 1 M H_2_SO_4_ using a Tecan infinite F200pro plate reader (Tecan, Männedorf, Switzerland). At least three independent sets of experiments were performed. The biological replicates of each set were then normalised to control to allow for combined statistical analysis.

### 4.7. Real Time RT-PCR

Total RNA from HUVECs was isolated as previously described [30], reverse transcribed and analyzed as outlined previously [31,32]. We analysed mRNA expression with an ABI 7500 Real-Time PCR System (Thermo Fisher Scientific). Quantification was done according to the manufacturer’s instructions using pre-made probes for DICER1 (Hs00229023_m1) and DROSHA (Hs00203008_m1). As endogenous control, Hypoxanthine-guanine phosphoribosyltransferase 1 (HPRT1; Hs02800695_m1) was used. We performed relative quantification of gene expression using the delta-delta Ct method [33].

### 4.8. Western Blotting

Western Blot analyses were performed as previously described [31,34] with endothelial cells lysed using commercial lysis buffer (Cell Signaling Technology, Danvers, MA, USA) supplemented with a premade protease- and phosphatase-cocktail (Thermo Fisher Scientific). Cell debris were removed by centrifugation for 5 min at 15,000× *g*, 4 °C. SDS-PAGE was performed with equal amounts of protein per lane, followed by transfer to nitrocellulose membrane. Then, membranes were incubated with primary antibody solutions of DICER1 (Cell Signaling Technology; clone D38E7; 1:1000), DROSHA (Cell Signaling Technology; clone D28B1; 1:1000) and β-Actin (clone AC-15, Sigma Aldrich) according to the manufacturer’s instructions. Protein detection was achieved with peroxidase-conjugated secondary antibodies and the ECL system (Amersham Bioscience, Amersham, UK).

### 4.9. In Vivo Endothelial Spheroid Grafting Assay

For HUVEC xenograft analyses, male and female NOD-scid IL2rg^null^ (NSG^TM^) mice were used that carry two mutations on the NOD/ShiLtJ genetic background: severe combined immune deficiency (Prkdc^scid^) and a complete null allele of the IL2 receptor common gamma chain (IL2rg^null^). These mice were obtained from the Jackson Laboratory (stock # 005557) and were used in accordance with the directive 2010/63/EU and the German law (Tierschutzgesetz).

The *in vivo* endothelial spheroid grafting assay was carried out as described by Laib et al. [19]. HUVEC were transduced with lentiviral vectors to express specific shRNA directed against DICER1 or DROSHA or to express non-targeting shRNA control. Subsequently, spheroids of transduced HUVEC were generated using the hanging drop method. Endothelial spheroids were then embedded into a matrix containing endothelial growth medium, methocel, fibrinogen, growth factors (VEGF-A, bFGF) and high concentration matrigel (Corning). This suspension was injected subcutaneously into the ventral region of NSG^TM^ mice. For explantation of plugs, mice were sacrificed 21 days after injection. Plugs were immediately fixed in 4% formaldehyde and finally embedded in paraffin following standard protocols. Three 8 µm thick sections were sliced from different levels of the plug and humanoid neovessels were stained using fluorescently labelled Ulex Europaeus Agglutinin I (UEA I, Vector Laboratories, Burlingame, CA, USA) and analyzed morphometrically. For this purpose, eight images were acquired from each section and then analyzed using the Nikon NIS elements software. The values obtained in this way for the different images of the different sections of each plug were then averaged so that only one value was obtained for each plug and each parameter (blood vessel area, blood vessel density, average blood vessel size). In addition, phenotypic characterization of the vascular network derived from HUVEC was performed by immunohistochemistry using antibody labelling of human endothelial cell markers CD31 (Cell Signaling Technology; clone 89C2; 1:750) and CD34 (Thermo Fisher Scientific; clone QBEnd/10; 1:500). HRP-conjugated secondary antibodies were used as described by the manufacturer (Cell Signaling Technology, HRP-Rabbit (#8114); HRP-mouse (#8125)). For staining, we used the DAB-substrate kit according the manufacturer instructions (Cell Signaling Technology, #8059). Computer-aided histomorphometric analyses of humanoid neovessels were made using the NIS elements software package (Nikon).

### 4.10. Global Proteome Analyses, Protein Isolation and TMT Labeling

For TMT labeling experiments, HUVEC were transduced with lentiviral vectors for stable delivery of shRNA directed against DICER1, DROSHA or DICER1 and DROSHA or for stable expression of non-targeting shRNA control, respectively. 72 h post transduction, cells were washed, harvested, and lysed using commercial lysis buffer (Cell Signaling Technology) supplemented with a premade protease- and phosphatase-cocktail (Thermo Fisher Scientific). Samples were subsequently stored on ice and homogenised by sonication, after which protein concentrations were quantified by BCA analysis according to the manufacturer’s instructions (Thermo Fisher Scientific). Aliquots of cell lysates containing 100 μg total protein were taken and further processed for LCMS analysis following an adapted FASP (filter-aided sample preparation) protocol. Briefly, protein samples were incubated and washed with UA (8 M urea in 50 mM HEPES (4-(2-hydroxyethyl)-1-piperazineethanesulfonic acid), pH 8.5, 10 mM TCEP) in 0.5 mL Amicon Ultra centrifugal filter units (30 kD cutoff, Sigma Aldrich, Darmstadt, Germany) by centrifugation at 14,000× *g* for 2 × 10 min, alkylated with iodoacetamide (50 mM iodoacetamide in 8 M urea, 50 mM HEPES, pH 8.5) at RT for 20 min in the dark and washed twice with 50 mM HEPES, pH 8.5 (2 × 10 min at 18,000× *g*) before incubation with 40 µL of trypsin (2 µg) in 50 mM HEPES, pH 8.5 at 37 °C overnight. Digests were labeled with TMT 10plex isobaric labels (Thermo Fisher Scientific) according to the manufacturer’s protocol before the differently labeled samples were combined for subsequent analyses.

### 4.11. LC–MS/MS Analysis

Peptide solutions were analyzed by liquid chromatography/tandem mass spectrometry (LC-MS/MS) on Ultimate 3000 RSLC nano-HPLC systems coupled to an Orbitrap Fusion mass spectrometer with a an EASY-Spray™ ion source (all from Thermo Fisher Scientific). Samples were loaded onto an RP C18 pre-column (Acclaim PepMap, 300 μm × 5 mm, 5 μm, 100 Å, Thermo Fisher Scientific) at a flow rate of 30 μL/min and washed with 0.1% (*v*/*v*) TFA for 15 min at 30 μL/min before elution and separation on a self-packed RP C18 separation column (PicoFrit, 75 μM × 250–500 mm, 15 µm tip diameter, packed with *ReproSil-Pur* C18-AQ, 1.9 μm, 120 Å, Dr. Maisch, Ammerbuch, Germany) equilibrated with 3% solvent B (solvent A: 0.1% (*v*/*v*) FA (formic acid), solvent B: ACN, 0.08 % (*v*/*v*) FA). A gradient from 3–40% solvent B within 360 min at 300 nL/min was used to elute peptides from the separation column. Data were acquired in data-dependent MS/MS mode using stepped HCD (high energy collisional dissociation, normalised collision energies (NCE): 30, 35, 40%) and CID (collisional induced dissociation, NCE: 35%) for fragmentation. For data acquisition, each high-resolution full scan (*m*/*z* 300 to 1500, R = 120,000, target value (TV) 400,000, max. injection time (IT) 50 ms) in the orbitrap was followed by high-resolution (R = 60,000, TV 50,000, IT 50 ms, isolation window 1.3 Th) in the orbitrap and low resolution product ion scans in the linear ion trap (TV 10,000, IT 50 ms, isolation window 2 Th) for charge states 2+ to 7+ within 5 s, starting with the most intense signal in the full scan mass spectrum. Dynamic exclusion (duration 45 s, window ± 2 ppm) was enabled. The Xcalibur software (version 4.1, Thermo Fisher Scientific) was used for data acquisition.

### 4.12. MS Data Analysis

For peptide identification, MS/MS data were searched against the Uniprot database (UP000005640, Taxonomy Homo sapiens, 78,120 entries) using the Byonic algorithm (version 2.16.2 Protein Metrics Inc., Cupertino, CA, USA). A maximum mass deviation of 15 ppm was applied for precursor ions, whereas for product ions, max. 0.6 Da (linear ion trap data) and 30 ppm (orbitrap data) were allowed. Oxidation of Met, acetylation of protein N-termini, and phosphorylation of S, T and Y were set as variable modifications, and carbamidomethylation of cysteines modification of peptide N-termini as well as modifications of Lys by the TMT label were included as fixed modifications. A maximum of two missed cleavage sites were considered for peptides and allowed for semi-specific digestion. Automatic score cut for peptide output and protein FDR was left for downstream processing (as recommended). Individual results files were exported as mzIdentML files with associated MGF spectra files, and imported into Scaffold 5 (Proteome Software Inc., Portland, OR, USA). Proteins were accepted at 95% FDR threshold for quantification. Quantification was performed using the TMT reporter ion ratio derived from the FT HCD spectra.

### 4.13. Statistical Analyses

Statistical analyses were performed using one-way analysis of variance followed by the Bonferroni’s Multiple Comparison test, by the unpaired student‘s t-test or by the Mann–Whitney-U-Test. For statistical analyses the Graph Pad Prism 6 software package was used (Graph Pad Software, Inc., La Jolla, CA, USA). Data were expressed as mean ± standard deviation (SD) or as indicated otherwise. Statistical analyses within Scaffold comprised a Mann–Whitney Test with a corresponding Benjamini–Hochberg multiple comparison test (*p* values calculated for each experiment internally) and data filtered accordingly for downstream analyses. Further statistical analyses, such as a heatmap, were performed using R (3.6.0). All *n* values represent independent biological replicates. Probability values were considered significant at a *p* < 0.05.

## Data Availability

The data that support the findings of this study are available within the article or from the corresponding author upon reasonable request. The raw mass spectrometry data obtained were deposited on the ProteomeXchange Consortium (http://proteomecentral.proteomexchange.org on (accessed on 13 August 2021)) with the dataset identifier PXD027923.

## Supplementary Materials

The following are available online at https://www.mdpi.com/article/10.3390/ijms22189855/s1.
