# Supplementary material for: Impact of DICER1 and DROSHA on the Angiogenic Capacity of Human Endothelial Cells
Source: Int J Mol Sci. 2021 Sep 12;22(18):9855. doi: 10.3390/ijms22189855 (PMC8471234; doi:10.3390/ijms22189855)
Supplement: Supplementary file 1 [file ijms-22-09855-s001.zip › ijms-1368675-supplementary.pptx]

## Slide 1
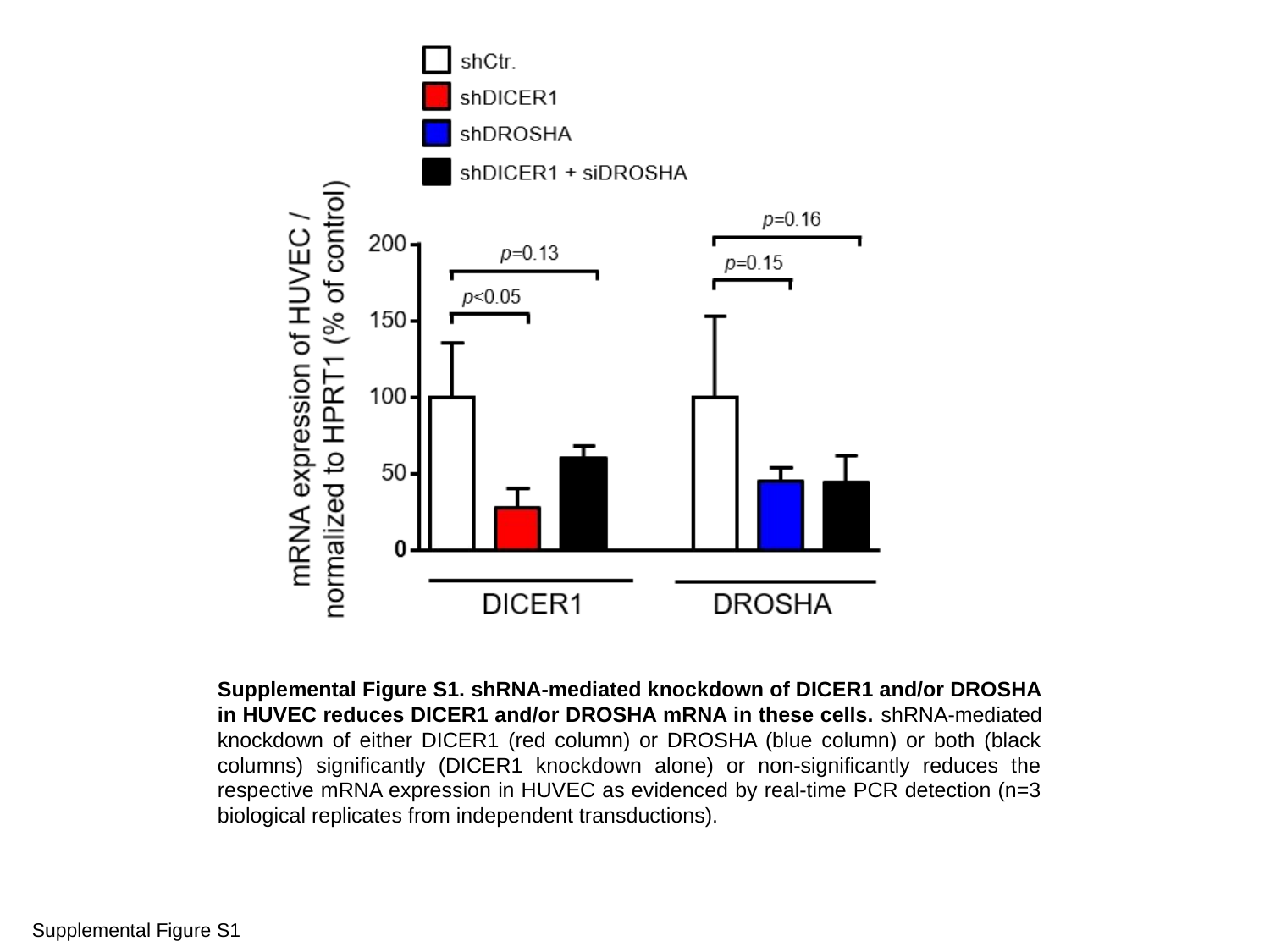

Supplemental Figure S1. shRNA-mediated knockdown of DICER1 and/or DROSHA in HUVEC reduces DICER1 and/or DROSHA mRNA in these cells. shRNA-mediated knockdown of either DICER1 (red column) or DROSHA (blue column) or both (black columns) significantly (DICER1 knockdown alone) or non-significantly reduces the respective mRNA expression in HUVEC as evidenced by real-time PCR detection (n=3 biological replicates from independent transductions).
Supplemental Figure S1
